# Potential Use of *Biomphalaria alexandrina* Snail Antigens for Serodiagnosis of Schistosomiasis Mansoni by Immunoblot Analysis

**Published:** 2013

**Authors:** Maha MA Basyoni, Azza Abd EL-Wahab

**Affiliations:** 1 Dept. of Parasitology, Faculty of Medicine, Cairo University, Cairo, Egypt; 2 Dept. of Parasitology, Faculty of Veterinary Medicine, Cairo University, Cairo, Egypt

**Keywords:** Schistosomiasis *mansoni*, Snail antigens, EITB

## Abstract

**Background:**

The aim of this study was to evaluate the possible use of *Biomphalaria alexandrina* snail antigens in diagnosis of schistosomiasis mansoni using enzyme linked immunolectrotransfere blot (EITB).

**Methods:**

*S. mansoni* adult worm crude antigens (AWA), feet and visceral humps of *B. alexandrina* and *Bulinus truncatus* were used. Hyperimmune mice sera (HIS) versus each antigen were prepared for diagnosis of *S. mansoni* using western blot (WB).

**Results:**

Snail foot antigens were more specific in antibodies detection than visceral hump antigens. Three of five polypeptides of *B. alexandrina* foot antigen identified by *S. mansoni* HIS showed specific positive reactivity. These polypeptides were at MW of 31/32 and 43 kDa. While, only one of the six polypeptides of *B. alexandrina* hepatopancrease antigen identified by *S. mansoni* HIS, at a MW of 43 kDa was specific. Similarly, 2 polypeptides at MW of 44 and 55 kDa were specific in detection of anti- *S. haematobium* antibodies. However, the antigenically active polypeptide of *B. truncatus* hepatopancrease antigen had no specific reactivity towards anti-*S. haematobium* antibodies.

**Conclusion:**

*B. alexandrina* foot antigens were the most specific of the tested snail antigens in diagnosis of schistosomiasis *mansoni*.

## Introduction

Despite widespread efforts for schistosomiasis management, it remains a main health dilemma in tropical and subtropical districts (1). Serodiagnosis is essential for diagnosis of parasitic diseases with its precision being affected by types and extent of purification of the used antigens (2). Sodium dodecyl sulphate polyacrylamide gel electrophoresis (SDS-PAGE) as a method of purification, overcome the cross-reactivity between diverse antigenic components and augmented the antigenic yields by increasing the number of antigens which might be present in little amounts (3). Newly emerging antigenic fractions strongly linked to human species were found specific diagnostic tools. This is evident in schistosomiasis where antigenic sharing between adults, immature stages and intermediate hosts was established (4).

Establishment of trematodes infection in their definitive hosts is confirmed by demonstration of certain circulating antibodies detected against antigens of their snail intermediate hosts (5).

Sera of patients infected with *S. mansoni* demonstrated antibodies against hepatopancrease of *B. glabrata* snails infected with homologous cercaria. The researchers explained that the glycocalyx of *Schistosoma* cercariae contains antigens contracted from *B*. *glabrata* snail tissues (6). Sulahian et al. (7) considered that using EITB is an accurate mean of detection of specific *Schistosoma* antibodies.

The current study was directed to detect the value of *B. alexandrina* snail antigens in diagnosis of schistosomiasis *mansoni* using EITB.

## Materials and Methods

### Preparation of snail antigens

*B. alexandrina* and *B. truncatus* medium to large sized snails were collected from Abu-Rawash, Giza and reared in the laboratory for production of laboratory- bred snails according to EL-Bahy (8).^.^After 4-6 weeks snails were dissected into two parts, a foot and visceral hump. These were separated, homogenized, sonicated and left overnight for extraction (9).The protein content of the prepared antigens was measured (10), divided into aliquots and stored at -20 °C until used.

### Preparation of Schistosome adult worm crude antigen

Cercariae of *S. mansoni* and *S. haematobium* (Egyptian strain) were provided by Schistosome Biological Supply Program Unit (SBSP), Theodor Bilharz Research Institute, Giza, Egypt. They were obtained from experimentally infected *B. alexandrina* and *B. truncatus* snails. Twenty to thirty cercariae were injected intra-peritoneal in each Swiss albino mice (22 mice, 15-20 gm each) and 8 weeks later, adult worms were collected from liver and premesenteric veins (11). They were homogenized, sonicated then centrifuged at 20000 rpm for 1 hour at 4 °C. The supernatant containing the crude antigens was distributed into 1ml aliquots in plastic vials after measuring its protein content as above and stored at -20 °C until used (12).

### Preparation of specific hyper-immune mice sera

Mice HIS were prepared against snail and parasite antigens according to Langly and Hillyer (13) via initial subcutaneous injection of an equal volume of Freund ’s complete adjuvant mixed with 0.4 mg protein for each antigen and injected subcutaneously at different places at back of mice. After 3 weeks, another 0.4 mg of protein for each antigen, mixed in an equal volume of Freund ’s incomplete adjuvant and divided into 3 doses, injected intramuscularly at biweekly intervals. Ten to fourteen days later, the blood was collected from retro**-**orbital vein using capillary tubes under ether inhalation anesthesia. Serum separation was obtained by centrifugation at 3000 rpm for 5 minutes. The level of specific antibodies in sera of immunized mice was evaluated. Negative control sera were obtained by bleeding mice prior to immunization.

### SDS-PAGE and electrophoretic transfer

The protein components of *S. mansoni* adult crude worm antigens and snails’ antigens were separately resolved by SDS-PAGE under reducing conditions using 12% SDS-PAGE (100µg/Lane) (Bio Rad System; USA) according to Laemmli (14). After the electrophoresis, the resolved polypeptides were electrophoretically transferred to a nitrocellulose membrane for immunoblotting using a transfer apparatus (15).The antigen-blotted nitrocellulose membrane was cut vertically into strips of 15x0.5 cm. One strip was incubated with one serum sample diluted 1:50 in 2 ml blocking solution (1% skimmed milk in 0.1 M phosphate buffered saline, pH 7.4, containing 0.0.2% Tween 20 (EL Gomhoreya pharmaceutical CO.) for 2 h at room temperature (RT) with gentle rocking, washed five times with blocking solution, and then incubated for 2 h at RT with peroxidase conjugated goat anti-mouse IgG antibody. The conjugate was used at a concentration of 1:500 in blocking buffer (Sigma, Immunochemical). After washing, the strips were incubated in (amino-9-Ethyl carbazole; AEC) for 30 minutes. The strips were then washed with distilled water to stop the reaction, air-dried and examined for color development at the site of positive reacting fractions using software Gel pro-analyzer.

## Results

**Fig. 1:**
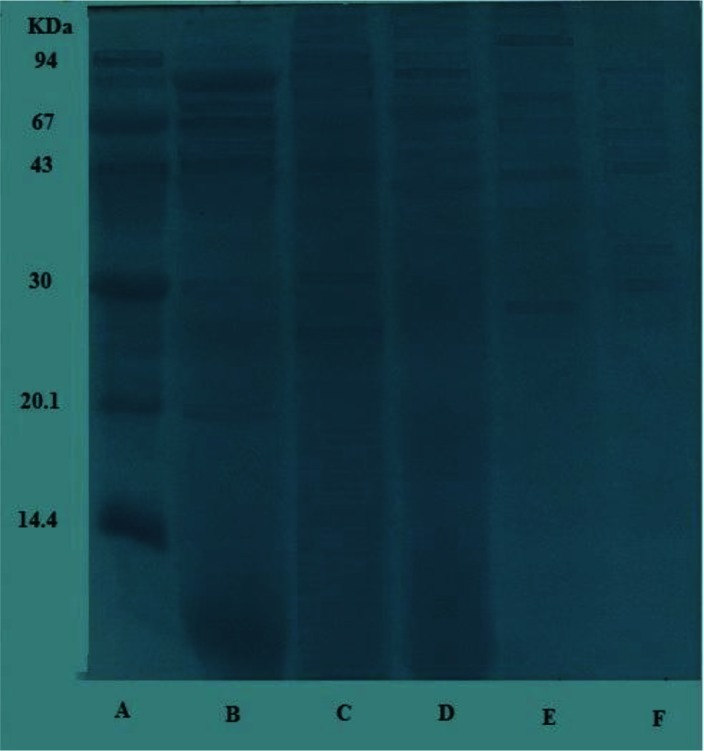
SDS-PAGE fractionation of the tested antigens A: Low molecular weight standard B: Fractioned *S. mansoni* adult worm crude antigen C. Fractioned *B. alexandrina* foot antigen D: Fractioned *B. alexandrina* visceral hump antigen E: Fractioned *B. truncatus* foot antigen F: Fractioned *B. truncatus* visceral hump antigen

Fractionation of *S. mansoni* snail and adult worm crude antigen revealed multiple components via SDS-PAGE. Adult *S. mansoni* crude antigen revealed at least 9 polypeptides. Those polypeptides molecular weights ranged from16-120 kDa. Of them, 7 protein bands corresponding to molecular weights (MW) of 92, 70, 67,54,44,30 and 20 kDa were the most prominent. Concerning *B. alexandrina* foot antigen, it revealed the presence of 11 polypeptides in each antigen. Their MW ranged from 20 to 115 kDa. The major protein bands were at MW of 109,102,94,69,54,50,32,28 and 22 kDa. While that of *B. alexandrina* visceral hump antigen revealed the presence of 12 visible protein bands. Their MW ranged from 13 to 110 kDa. The major bands were at 103, 94, 93, 85, 69, 64, 54, 50, 36.5 and 33 kDa. *B. truncates* foot antigen revealed 5 major protein fractions at MW of 98,71,60,43 and 29 kDa while that of *B. truncatus* visceral hump antigen revealed 7 major protein fractions at MW of 90, 72.5, 61.5, 55, 47.5, 36 and 31 kDa (Fig. 1).

**Fig. 2:**
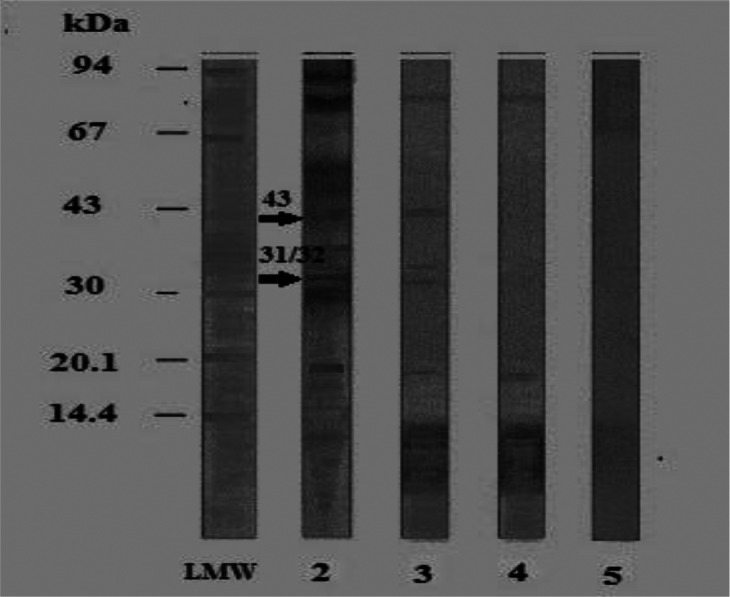
Immunoblot reaction of the tested *B. alexandrina* foot antigen using homologous and parasite hyperimmune mice sera (dilution 1:50) LMW: Low molecular weight standard 2: Specific protein bands of *B. alexandrina* foot antigen reacted against homologous HIS 3: Reacted *B.alexandrina* foot antigen against *S. mansoni* HIS/4: Reacted *B.alexandrina* foot antigen against *S.haematobium* HIS/5. Reacted *B.alexandrina* foot antigen versus negative mice sera (negative control)

Data in Fig. 2, revealed that antigenically active components in *B. alexandrina* foot antigen on reaction with its homologous HIS, 9 polypeptides at MW of 77/76, 43, 36/37,31/32,20 and 14 kDa were detected (Fig. 2,Lane 2). All those polypeptides except that of 36/37 and 77/76 kDa were recognized by *S. mansoni* HIS (Fig. 2, Lane 3).While *S. haematobium* HIS reacted crossly with 2 polypeptides at MW of 20, 14 kDa (Fig. 2, Lane4). No polypeptides were identified by pre-immunized mice sera (Fig. 2, Lane 5).Therefore, the polypeptides at MW of 31/32 and 43 kDa were considered to be specific in detection of *S. mansoni* antibodies.

**Fig. 3:**
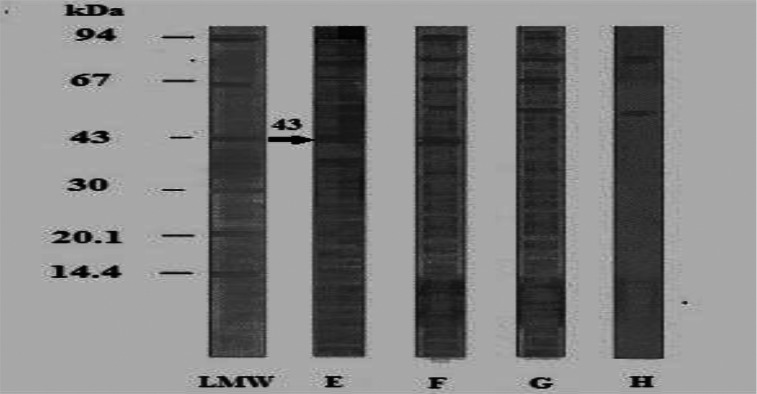
Immunoblot reaction of the tested *B. alexandrina* visceral hump using homologous and parasite hyperimmune mice sera (dilution 1:50) LMW: Low molecular weight standard 2: Specific protein bands of *B. alexandrina* visceral hump antigen reacted against homologous HIS 3: Reacted *B.alexandrina* visceral hump antigen against *S. mansoni* HIS 4: Reacted *B.alexandrina* visceral hump antigen against *S.haematobium* HIS 5. Reacted *B.alexandrina* visceral hump antigen versus negative mice sera (negative control)

*B. alexandrina* visceral hump antigen revealed 7 antigenically active polypeptides at MW of 93,80,70,55,52,43 and 40KDa(Fig. 3,Lane 2).

**Fig. 4:**
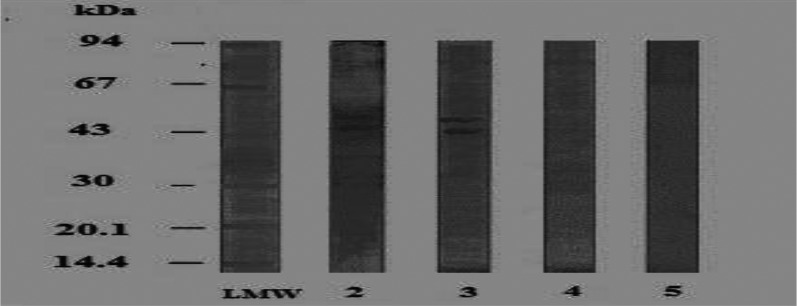
Immunoblot reaction of the tested *B.truncatus* foot using homologous and parasite hyperimmune mice sera (dilution 1:50 LMW: Low molecular weight standard 2: Specific protein bands of *B.truncatus* foot antigen reacted against homologous HIS 3: Specific protein bands of the reacted *B.truncatus* feet antigen against *S.haematobium* HIS 4: Non -reacted *B.truncatus* feet antigen against *S. mansoni* HIS. 5. Non-reacted *B.truncatus* feet antigen versus negative mice sera (negative control)

All of them were recognized by *S.mansoni* HIS except that at 40 kDa (Fig. 3, Lane 3). From these polypeptides, five at MW of 93,80,70,55,52 reacted crossly with *S. haematobium* HIS (Fig. 3, Lane 4). Pre-immunized mice sera recognized polypeptides at MW of 55 and 80 kDa (Fig. 3, Lane 5). Thus only one polypeptide at MW of 43 kDa was specific in detection of anti-*S. mansoni* antibodies. Concerning *B. truncatus* foot antigen, its homologous HIS recognized bands at 44 and 55 kDa (Fig. 4, Lane 2).

**Fig. 5:**
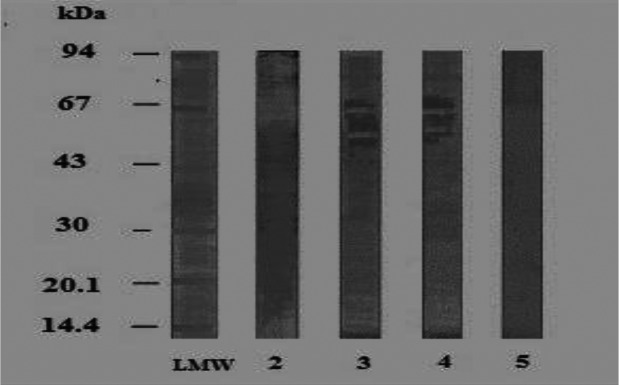
Immunoblot reaction of the tested *B. truncatus* visceral hump antigens using homologous and parasite hyperimmune mice sera (dilution 1:50) showing no specific reactivity of *B. truncatus* visceral hump antigen against homologous HIS (Lane 2), similar non-specific reactivity of *B. truncatus* visceral hump antigen against *S. haematobium* and *S. mansoni* HIS (Lanes 3&4) and non-reactivity against pre-immunized mice sera (Lane 5).

Both polypeptides were identified by *S. haematobium* HIS (Fig. 4, Lane 3). It did not react crossly with *S. mansoni* HIS (Fig. 4, Lane 4) or with pre-immunized mice sera (Fig. 4, Lane 5). Thus, 2 polypeptides at MW of 44 and 55 kDa were specific in detection of anti- *S. haematobium* antibodies. On the other hand, the antigenically active polypeptide of *B. truncatus* visceral hump antigen had no specific reactivity towards anti*-S. haematobium* antibodies (Fig. 5, Lane 2). Similar antigenic profiles were observed with both *S. haematobium* HIS (Fig. 5, Lane 3) and *S. mansoni* HIS (Fig. 5, Lane 4). No cross reactivity with pre-immunized mice sera was detected.

## Discussion

In tropical and subtropical regions, schistosomiasis attains a chief health concern (16). In Egypt, the Nile valley area is highly endemic for schistosomiasis with infection rates beyond 80% bringing Egypt to be one of the most endemic countries in the world (17).

Researches based on developing new antigens for diagnosis of schistosomiasis focused on improving the extent of sensitivity and specificity of the selected antigen which in turn enhanced the accuracy of the serological test used (3).

The possibility of the use of shared antigens between trematodes and their intermediate hosts for serodiagnosis is apparent in schistosomiasis depending on the described antigens shared between schistosomes and their intermediate hosts (18). The current study was directed to detect the value of Schistosome snail antigens in diagnosis of schistosomiasis *mansoni* using EITB. The antigenic community between snails and their trematode parasites was already investigated and it was suggested that molecular mimicry of some of the shared antigens manifests a genetic accommodation between the parasite and its host (19).

In our study a variety of antigens were prepared from *S. mansoni* adult worms and their snail intermediate host (*B. alexandrina* foot and visceral hump antigens) and from *S. haematobium* adult worms and their snail intermediate host (*B. truncatus* visceral hump and foot antigens). They were used to clarify the antigen antibody relation between schistosomes and their related snails.

The current study clarified the antigenic similarity between *S. mansoni* and its snail intermediate hosts from the aspect of molecular weights using SDS-PAGE and from the aspect of immune reaction using WB technique after preparation of HIS. Our results revealed the presence of 7 major protein bands of the fractioned *S. mansoni* AWA corresponding to molecular weights of 92, 70, 67,54,44,30 and 20 kDa.

These results showed some similarity to Rupple et al. (20) who reported that in all chronically old infected mice, antibodies against *S. mansoni* AWA proteins of 67 kDa were prominent.

When sera of *S. mansoni* infected patients were analyzed by immunoblotting during the prepatent infection, anti- adult IgG antibodies elucidated an intense reaction recognizing the area of 30-40 kDa (21). In addition, Soliman et al. (22) used SDS-PAGE to analyze soluble worm antigens, cercarial antigen preparations and soluble egg antigens of *S. mansoni*. The authors reported that 32 kDa was a chief band of these three fractionated antigens.

In the present work, we found that fractionation of *B. alexandrina* foot antigen revealed the presence of 9 major protein bands at MW of 109,102,94,69,54,50,32,28 and 22 kDa. While, that of *B. alexandrina* visceral hump antigen revealed the presence of 10 major protein bands at 103, 94, 93, 85, 69, 64, 54, 50, 36.5 and 33 kDa. These results showed that the AWA and both *B. alexandrina* antigens have a common polypeptide band at MW of 54 kDa.

When comparing the same snail species, Shalaby et al. (23) reported that *B. alexandrina* snail foot and hepatopancrease antigens contained 8 and 7 polypeptides by SDS-PAGE respectively. Their molecular weights ranged from 21-97 kDa. They added that the component of 97 and 30 kDa were major bands in *B. alexandrina* foot while, that of 66 and 30 kDa were major bands in *B. alexandrina* hepatopancrease.

The potential of *B. alexandrina* antigens was studied by Gamal-Eddin et al. (24) who concluded that immunization of albino mice with purified F_iv_ 20-29 kDa &F_v_ 20-24 kDa antigenic fractions of *B. alexandrina* resulted in reduction of the worm burdens, the total eggs in tissues and reduced the histopathological changes in the liver.

The MWs of these polypeptides are a little bit different from the ranges of MWs of foot and visceral extracts revealed by our study, which may be attributed to differences in the antigen preparations, techniqual differences, use of different concentrations of the resolving gel and different concentration of proteins by different researchers.

In the present study we evaluated the specificity of *Schistosoma* snail foot and visceral hump antigens in antibody detection of *S. mansoni* by evaluating their reaction versus specific prepared HIS at a dilution of 1:50 via WB technique.

Our results indicated that *B. alexandrina* foot antigen was more specific than *B. alexandrina* hepatopancrease antigen in antibody detection. Where, three of five polypeptides of *B. alexandrina* foot antigen identified by *S. mansoni* HIS showed specific positive reactivity. These polypeptides were at MW of 31/32 and 43 kDa. While on the other hand, only one of the six polypeptides of *B. alexandrina* hepatopancrease antigen identified by *S. mansoni* HIS, at a MW of 43 kDa was specific. Moreover, several molecules were inhibited as 77/76 and 36/37 kDa components that failed to be recognized by *S. mansoni* HIS. Also, cross inhibition detected by failed recognition of 77/67, 36/37, 31/32 and 43 kDa molecules by incubation of *B. alexandrina* foot antigen with *S. haematobium* HIS, reinforcing the hypothesis of antigenic community between *S. mansoni* and *B. alexandrina* foot antigen.

Coinciding with our results, when immunoblotting tests employed to detect AWA antibodies, an immunogenic fraction with a molecular weight of 31-32 kDa (Sm 31/32) was considered to be the most frequently documented fraction. It revealed 98% positivity in the pre- treatment stage then declined or became negative in the serum of treated patients exposed to low infection. Thus it is considered of high diagnostic importance and could therefore be used as a serologic marker (25–26).

These studies together with our data specifying 31/32 kDa band may give an evidence that *B. alexandrina* foot antigen is probably a suitable antigen to replace AWA during immunodiagnosis of schistosomiasis *mansoni*.

We reported no cross reactivity of *B. truncatus* feet antigen against *S. mansoni* HIS. In addition, no specific reactivity had been shown by antigenically active polypeptides of *B. truncatus* visceral hump antigen.

The specificity of *B. alexandrina* foot antigen to their trematodes was studied by Shalaby et al. (23) who described the specific reactivity of 54 and 45 kDa polypeptides fractionated from *B. alexandrina* foot antigen against anti-*Paramphistomum* antibodies. However, *B. alexandrina* hepatopancreases antigens had no specific reactivity towards homologous antibodies.

However, Rasmussen et al. (5) elucidated the widespread cross-reactivity of snail antigens with their incompatible trematodes by characterization of *S. mansoni* adult worm antigens obtained by affinity chromatography through an anti-*B. glabrata* column and reacted strongly with sera of humans and mice infected with *F. hepatica*.

Our experiment provides a support to the assumption of the antigenic community between *S. mansoni* and one of its vectors, *B. alexandrina*, through exhibition of common antigenic epitopes with its intermediate host.

*B*. *alexandrina* has the advantage of being an affluent source of antigens for serodiagnostic and prophylactic studies for *S*. *mansoni*. It is easy maintained in the laboratory, with a high protein yields and low expenditure, compared to other parasite antigens, as AWA and soluble egg antigens that require completion of the long life cycle. However, the cross-reactivity exhibited between the parasite and the mollusk restricted the specificity of these antigenic epitopes.

## Conclusion

*Biomphalaria alexandrina* foot antigen was the best antigen among the tested ones that can replace *S. mansoni* adult worm crude antigen thus, proved to have the potential as a screening tool for schistosomiasis immunodiagnosis. However, its sensitivity may necessitate more enhancements by purification of the used antigen. Moreover, the recognition and categorization of these antigenic epitopes is vital for the development of recombinant proteins that could be used in immunodiagnosis of schistosomiasis.Further trials are required in order to better characterize the cross-reactive and non cross- reactive snail antigen epitopes that are preferentially recognized by resistant experimental animals and humans.
